# Containerized cloud-based honeypot deception for tracking attackers

**DOI:** 10.1038/s41598-023-28613-0

**Published:** 2023-01-25

**Authors:** V. S. Devi Priya, S. Sibi Chakkaravarthy

**Affiliations:** Centre of Excellence, Artificial Intelligence and Robotics (AIR); Centre of Excellence, Cyber Security and School of Computer Science and Engineering, VIT-AP University, Amaravati, Andhra Pradesh 522237 India

**Keywords:** Engineering, Mathematics and computing

## Abstract

Discovering malicious packets amid a cloud of normal activity, whether you use an IDS or gather and analyze machine and device log files on company infrastructure, may be challenging and time consuming. The vulnerability landscape is rapidly evolving, and it will only become worse as more and more developing technologies, such as IoT, Industrial Automation, CPS, Digital Twins, etc are digitally connected. A honey trap aids in identifying malicious packets easily as, after a few rapid calibrations to eliminate false positives. Besides analyzing and reporting particular invasion patterns or toolkits exploited, it also assists in preventing access to actual devices by simulating the genuine systems and applications functioning in the network thus delaying as well as baffling the invader. In order to analyze and evaluate the hackers’ behavior, an ensemble of research honeypot detectors has been deployed in our work. This paper delivers a robust outline of the deployment of containerized honeypot deployment, as a direct consequence, these are portable, durable, and simple to deploy and administer. The instrumented approach was monitored and generated countless data points on which significant judgments about the malevolent users’ activities and purpose could be inferred.

## Introduction

As we navigate this brand-new digital revolution of massive heterogeneous data, difficult cyber security challenges started to crop up every day. It’s no surprise that cyber security issues are at their peak, and there’s a lot more to come in the future. It’s a significant concern how cyber-attacks are evolving in every way imaginable in order to stay on top of technological advancements. Phishing, crypto trojans, and cyber scamming are examples of common however threatening hacking attacks that actively pursue and take advantage of the user’s confidential information. Costs are dropped, productivity is raised, and network security is improved with a cloud-managed wireless network solution^1^. Internet and network fraudsters constantly attempt to compromise the security infrastructure of enterprises in order to grab sensitive information^2^. Hence being digitally secure becomes extremely challenging. The average cost of a data breach grew by 2.6 percent from 4.24 million USD in 2021 to 4.35 million USD in 2022^3^. From USD 3.86 million in the 2020 report, the average cost has increased by 12.7%. Further, it requires a lot of time to uncover a cyberattack. Attackers may be taking advantage of the system during this period while we are fully ignorant of it. By 2025, the global cybercrime impact is expected to hit 10.5 trillion dollars per year and digital security investment will surpass 1.75 trillion dollars^4^. This necessitates a need to investigate cyber security risks in order to effectively control the threat they pose. Intruders skilfully reap the benefits of exploitable vulnerabilities in a variety of methods that are generally difficult to pinpoint due to technological advancements. Therefore, it’s important to identify current shortcomings, how they can be used by adversaries in the manufacturing environment, and most crucially the facts they are most keen on^5^. The greatest approach to understand how to defend something is to be aware of what is going on within, which argues for security observability. In order to fully understand what is happening inside your API/microservices system and defend them from attack observability has to be employed^6^. Systems may be observed leveraging logs, statistics, and distributed traces, giving chance to spot intrusion attempts, fix security flaws, and prevent attacks before they can do significant harm. Many of these concerns are taken into account by Honeypots.

A honeypot offers information security groups broader visibility and empowers them to safeguard against assaults even the firewall can’t prevent. Many establishments worldwide incorporated them as an added layer for security from internal and external threats. A cyber honey trap lures intruders into a bait. It’s a computer system renounced to be attacked, or like a decoy designed to allure cyber attacks^2,7,8^. It imitates real phishing targets and leverages infiltration tactics to obtain intelligence about fraudsters and their methods of operation, or to divert them from all other aspirations. They might also have port numbers that seem to be accessible to a penetration test, which really is a method for determining whether ports on a node are vulnerable. The intruder may be lured by an open port, enabling the analyst to examine their attack pattern. Honeypot systems are exceptionally good at information collection, and they may also include signature-based intrusion detection, traffic acquisition, and internet protocol assessment, as well as flexible screening and fine-tuning. Honey-potting contrasts with other security techniques in that it is not aimed at preventing intrusions directly. It’s objective is to improve a firm’s intrusion detection mechanism and incident response so as to handle and mitigate assaults effectively. Anomalous and malicious traffic must be recognized in order for security personnel to analyze and restrict undesirable traffic flows in the communication network. Several machine-learning (ML) approach models that categorize harmful traffic flows using appropriate feature selection techniques^9–11^ has been presented by researchers in order to avoid fraudulent traffic flows in the network.

The significance of our Experimentation :

- Meticulously reviewed and evaluated the entire collection of information accumulated from the honeypot, whilst clearly demonstrating how to interpret them.
- Along with implying major motivations as well as alternatives for launching attacks, we could also provide a thorough rational reason for distinguishing botnet attacks.
- Successfully implemented a flexible polymeric decoy system that bestowed enthusiasts with a significant amount of information whilst retaining our prime goal, namely to create a prior warning entity.

The following is an overview of the manuscript formation: “Background” presents an outline of honeypot systems, virtualization & containerization, and precised the novelty of our experimentation, “Conceptual models of allied frameworks” sketches the conceptual models of allied security analysis frameworks, “Experimental setup” explored system experimental setup and its implementation details, “Analysis of the captured data” details the analysis of the captured data. Ultimately the paper concludes in “Conclusion and future enhancements”.

## Background

### Honeypot systems

Honeypots have the virtue of being targetable and hence facilitate the investigation of security vulnerabilities or the simulation of security vulnerability countermeasures^12^. Attacking honeypots is not regarded as a hazard as they often contain no vital or genuine information. Instead of acting as a standalone security mechanism like intrusion detection system, intrusion prevention system, and firewall, honeypots are regarded to be a component of surveillance systems, and the type of security mechanism required specifies how they’re being positioned.

**Figure 1:**
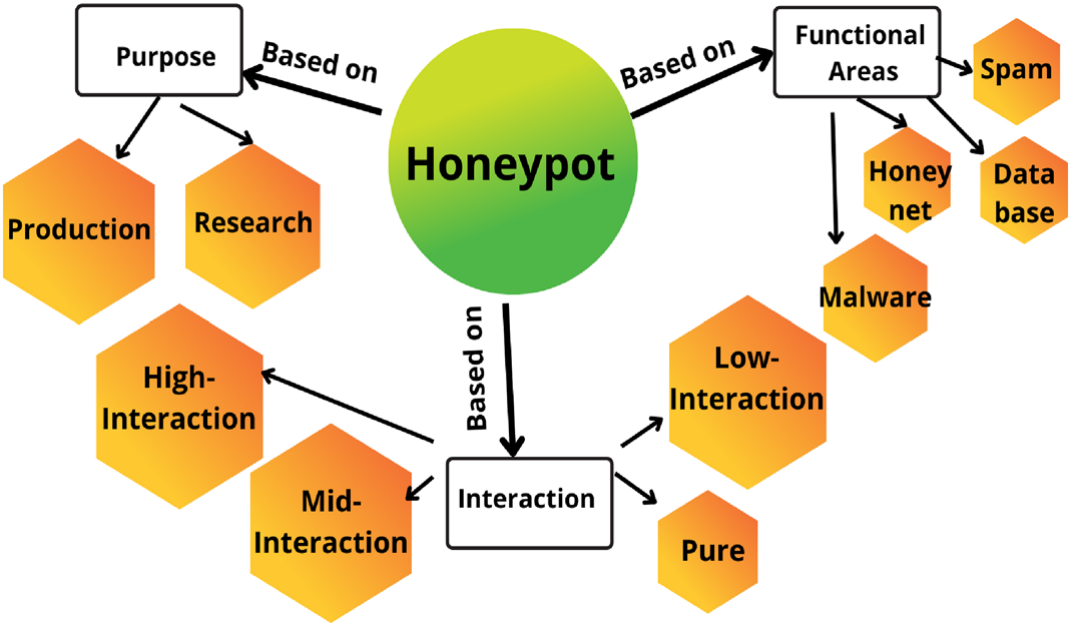
Categorization of honeypot systems.

Based on the purpose of honeypots (Fig. 1), they can be categorised as Production Honeypots and Research Honeypots^13,14^. Production honeypots are designed to detect intrusions in the intranet while also deceiving the malignant party. They play alongside real-world production workstations and provide the same functionality. They are commonly positioned close to surveillance assets in order to act as Indicators of Compromise (IoC) for both on-premise and external threats. Research honeypots gather information concerning intrusions, concentrating not only on how adversaries behave inside your own network but also on the broader population. Analyzing information and intelligence using honeypots can assist officials in developing robust defensive systems and determining which upgrades to activate. They are frequently positioned in a network’s Demilitarized Zone (DMZ) or that location where no operational activities occur (for example, via VLAN segmentation).

Honeypots are also categorized according to their degree of interactivity, which determines how an attacker performs intrusion on them as- High-interaction honeypot (Although they are not intended to replicate an overall production unit but do perform (or pretend to perform) most of the operations that a production process should, such as a complete operating system. The leveraging entity can observe adversary habits and strategies using this sort of honeypot), the Mid-interaction honeypot (These don’t have their own OS, but they do mimic features of the application layer protocol stack. They try to hinder or baffle adversaries so that enterprises get more time trying to sort out how to respond to an invasion), Low-interaction honeypot (These honeypots use fewer resources and collect only fundamental information on the type of risk or where it originated. They use TCP/IP (Transmission Control Protocol /Internet Protocol), and internet services and are reasonably straightforward to configure. There is, indeed, hardly anything within the honeypot that will keep the assailant’s focus for an extended period.eg: Honeyd^15^), Pure honeypot (These are large-scale, production-like system that runs around multiple servers. It is packed with sensors and holds “sensitive” information).

Depending on the functional area honeypots can be categorized as:- Malware honeypots (Identify malware by using established replication as well as exploitation channels.eg:-Ghost^16^), Spam honeypot(Unsecured proxies and email gateways are used in spamming honeypots to entice fraudsters. Spam traps can detect a spammer’s attempt and afterwards prohibit them from sending spam), Database honeypot (Create deception databases in order to entice database-specific exploits such as Sql injection attacks, which manipulate data in an unauthorized manner), Honeynets (comprised of multiple honeypots. Various assaults, such as DDoS (Distributed Denial-of-Service ) attacks, assaults on CDN( Content Delivery Network), and advanced persistent threats, can be examined by integrating various types of honeypots).

**Figure 2:**
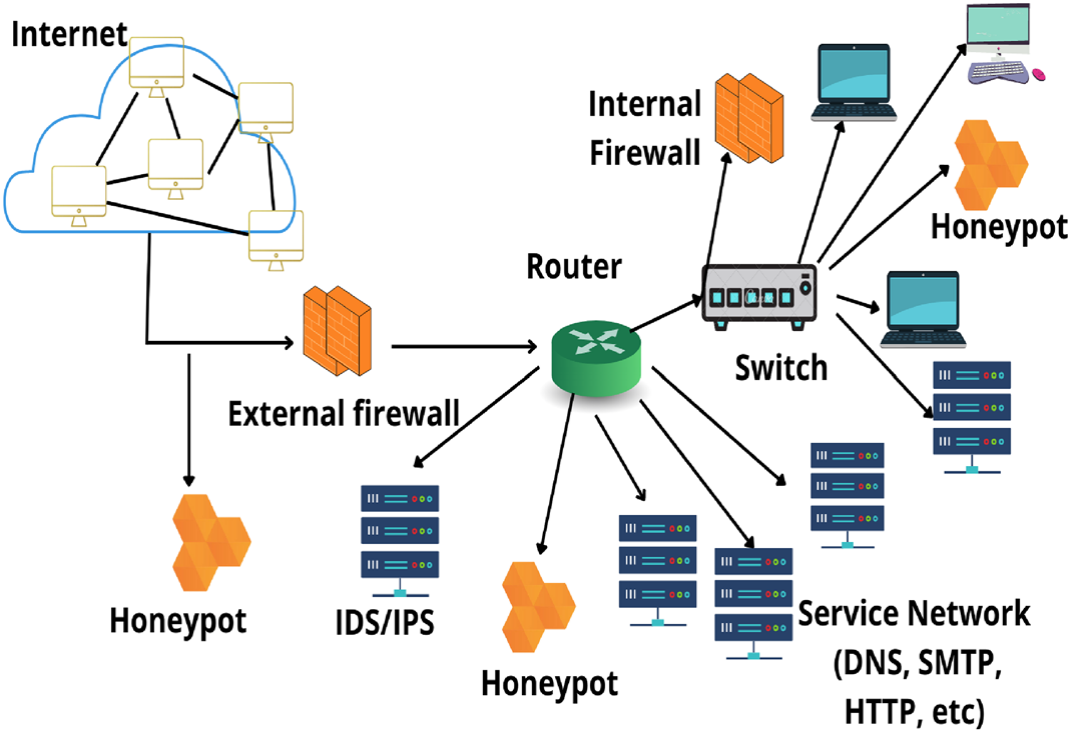
Honeypot system placement in an organizational network.

Depending on their intended application, honeypots can be planted both locally and externally. They are commonly installed in a demilitarized zone^17^ (DMZ; sometimes referred to as “perimeter network” or “screened subnet”) on the network. DMZ refers to a physical or logical subnetwork that uncovers a corporation’s external-facing services to an unsecured, typically bigger network, namely the World wide web. To track efforts to access the internal network, honeypots can be placed outside the external firewall and pointed at the internet. It can also be installed alongside the servers of a business network. Depending on the honeypot’s level of complexity, the type of traffic it hopes to draw, and how proximate it is to critical enterprise network assets, the honeypot’s exact positioning alters. Regardless matter where it is located, it will always be substantially clipped off from the production environment. The position is optimal in the three crucial areas of an organization, as demonstrated in Fig. 2^18^.

The major objectives of deploying honeypot can be enumerated as:

- To gather more about improbable threats and weaknesses.
- To serve as a predefined capture mechanism in which it draws assailants’ interest.
- To uncover fraudulent network activity.
- To provide a defense for actual systems by concealing them so that any attempt would hit the honeypots.
- To identify novel approaches and techniques (like zero-day attacks).

As compared to honeypots, firewalls are typically established over an organization’s perimeter to prevent unwanted accessibility by screening specific ports and content, however, they are not very effective at analyzing the traffic. An IDS or vulnerability detection system analyzes the communications and finds any improper, illegal, or unusual activities. But IDSs often experience the ’false warning issue’ as signature-based IDSs frequently produce false-negative warnings, whereas IDSs based on anomalies generate false -positive alerts^19^. The integration of IDSs with honeypot can greatly diminish faulty alerts.

### Virtualization and containerization

Most of those conventional methods for using honeypots become quite unproductive as engineers and technology professionals integrate containerization and microservices into their solutions. Containerization renders operating system-level virtualization^20^. The program and its prerequisites are packaged together in a container, which is a secluded virtual arena. As they share the firmware and relevant components with the OS, containers offer a lightweight solution as compared to virtual machines. Concise mono modules are deployed by microservices using containerization which together result in more flexible, extensible programs. This strategy eliminates the requirement to develop and release a completely novel version on each modification or scale of a particular function^21,22^.

### Novelty of our experimentation

Existing research^23^ on containerized honeypot data analysis has mostly focused on information pertaining to Industrial Control System (ICS) devices and is insufficient to analyze all of the data that has been collected. As the honeypot is deployed in the cloud, hackers could readily distinguish it as a decoy system. Hence, we strongly believe that the findings obtained were the repercussion of bots scanning the cloud and are therefore insufficient to improve the security intelligence of the ICS system as asserted by^23^.

Our exertion could distinctly demonstrate how to interpret data by critically analyzing and evaluating the whole evidence collected from our honeypot experimentation. We could also furnish detailed justification for identifying prevalent DDoS attacks while speculating on potential causes and alternatives. It is thus summarized that the virtue of absolute security can be accomplished by integrating honeypot data with IDS/IPS system. Moreover, the contributions asserted in this review further assist to recommence the areas of investigation in smart security intelligence.

## Conceptual models of allied frameworks

Since the year 2000, there were numerous distinct honeypot implementations, including fully accessible as well as commercial. Whilst some of them enable various services and emulation, others were built with a specific objective like protecting against a potential attack. Numerous authors worked in order to compile a list of honeypots.

An open and automated relay spamming research honeypot with Intellectual Simulated Analyzer (SpamPot)^24^ utilize Elasticsearch or OpenSearch to store data that has been filtered from received spam. It is comprised of two parts: the analyzer and the receiver. The receiver is a server (SMTP) that takes all emails sent on their way. The email is dumped in a directory together with its associated metadata, such as the IP address of the sender, the emails of the recipients, etc. Analyzer continuously keeps an eye on this directory in order to analyze these communications. The analyzer retrieves spams out from the receiver-shared directory and parses the .eml and metadata document. Information like URLs, attachments, mail body, etc. is extracted. If third-party extensions like VirusTotal, HatchingTriage, etc are set up with API keys, the indicators that have been retrieved from the email can then be queried through those applications. Elasticsearch then indexes this extracted data for subsequent searching and analysis. They still need to be integrated with SMTP verification and VirusTotal lookup.

A project called LaBrea^25^ makes a tarpit (or sticky honeypot) that establishes ’virtual computers’ that respond to connection requests by taking control of unoccupied Ipv4 on a local area network. The program assumes that the IP in concern is unused when it observes multiple successive ARP queries dispersed many clock cycles apart with no interim ARP response. Then it ’produces’ an ARP response and transmits it to the applicant with a fake MAC address. Valicek et al.^26^ proposes the use of a windows high interaction honeypot targeted for deployment in an office setting. The authors’ goal was a seamless integration of the project into a production setting.

In order to assess the present threat environment as well as how intelligent and crafty bad actors may be in compromising internet-facing ICS (Industry Control System), Rashid et al.^23^ implemented flexible low-interaction honeypot within AWS EC2 cloud instance over six distinct zones. Their research primarily focuses on honeypot data relevant to ICS systems and compares the top 10 AS/ASN attackers, interactions with various ICS devices and their geolocation, brute force attack rate, CVE and signature alerts, IP reputation, authentication events, various post-exploitation actions along with its sample instructions, malware signature, and interactions from different sources. Due to its installation in the cloud, hackers could easily distinguish the honeypot from a decoy. As a result, the information collected suspects to be produced by automated bots that scanned the cloud, and therefore insufficient to add on to the security intelligence of ICS systems. Methods that reduce the likelihood of a honeypot being discovered are mentioned in the work^27–30^.

## Experimental setup

Our experimentation is based on the works undertaken by DTAG (developed by Deutsche Telekom)^31^, which use an ensemble of docker containers along with an ELK stack for the honeypot administration and data analysis respectively. It uses docker as well as docker-compose to achieve its objective of executing a large number of tools concurrently and fully leveraging the hardware of the host. After successful implementation in the local device, the approach has been migrated to the cloud. This makes the overall mechanism relatively low maintenance as it enabled us to operate several honeypot sensors without any hassles along the same connection interface. Good segregation of the sandboxes and simple updating procedures are enabled by the docker encapsulation of the honeypot daemon.

The setup is carefully and consciously made to look like a valid target, mirroring the modeling approach of its components, structure, and content. This is done to persuade the opponent that they have gained access to the real system and to tempt them to stay. The program’s inbound traffic has been configured to accept all traffic. All persistent data files from the ensemble of honeypots can be found in the folder/data. Data logs related to each honeypot can be found at /var/lib/docker/containers/*/*.log. Filebeat serves as the logging agent or log data shipper, a light-weight shipper, installing itself on the system collecting the system logs, tailing them, and transmitting the information to Logstash for more sophisticated processing. Filebeat initiates one or more inputs that search for log data in the folder designated and launches a harvester for each log it discovers^32^. Whenever the harvester scans a single log for fresh data, it transmits the fresh log data to libbeat, which aggregates the events and delivers the aggregated data to the output that is set up for Filebeat. It was created in the Go language and has been optimized to handle massive amounts of data, support cryptography, and effectively withstand rear pressure. A freeware data engine entitled Logstash (written in Ruby) is used to ingest, process data from multiple viewpoints and outputs the data through the output plug-in to Elasticsearch. All log content is patched up, transformed, and given advanced downstream visualization and analytical depth^33^. The three steps of the Logstash event processing pipeline include inputs, filters, and outputs. Events are produced by inputs(beats/file/syslog/redis), modified by filters(grok/mutate/drop/clone/goip) , and sent there by outputs(elasticsearch/file). Both inputs and outputs accept codecs, letting you encrypt or decrypt data as it moves through the pipeline without the need for an additional filter. Regardless of format or complexity, it dynamically prepares relevant information like creating patterns from unstructured data, interpreting geographical information from IP addresses, removing all sensitive fields from personally identifiable data, and anonymizing it, regardless of the source of data, structure, or schema, simplifying overall processing.

**Figure 3:**
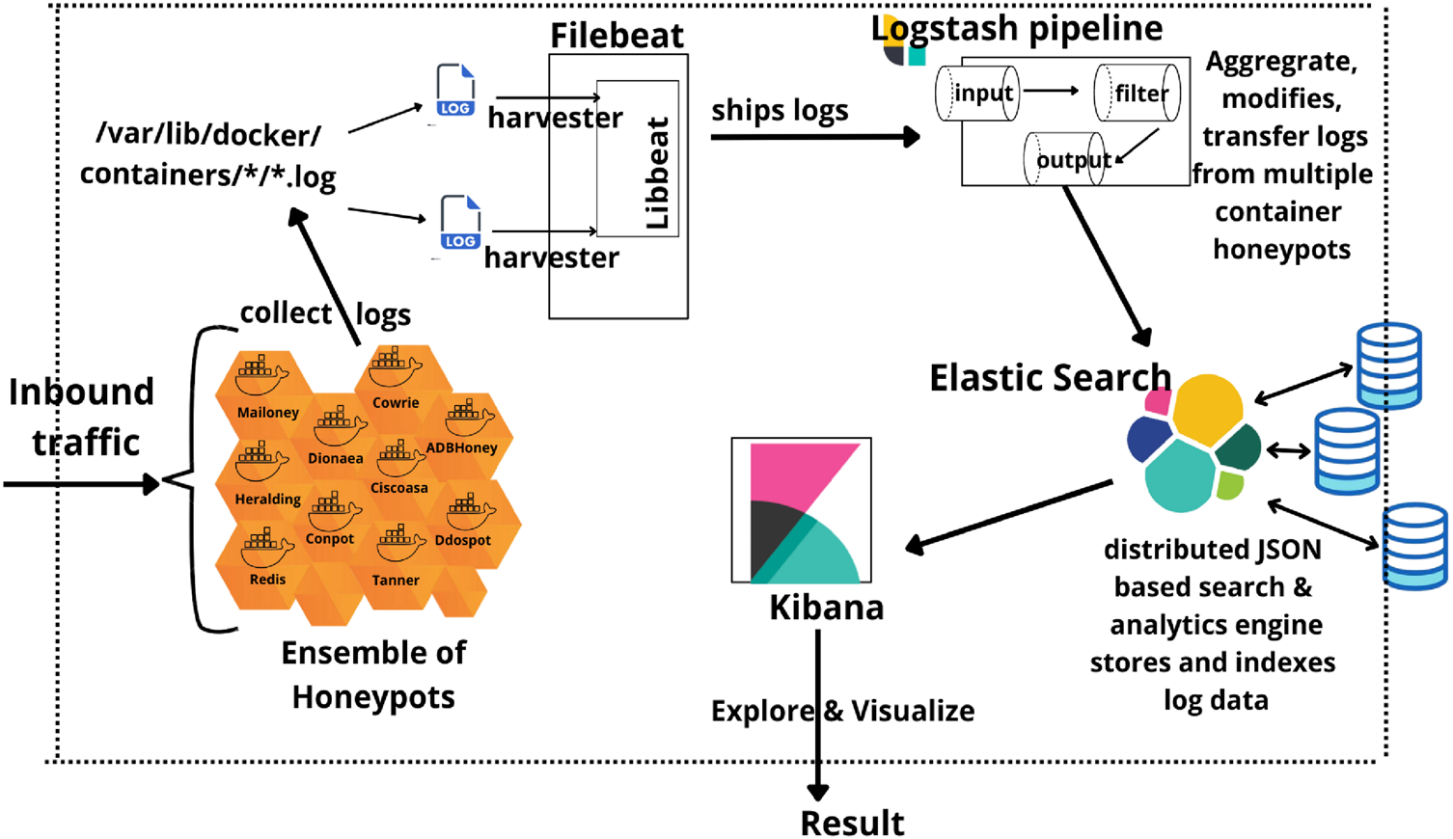
Collaborative operational testbed for real-time implementation using docker containerized honeypot.

Elasticsearch, a modern search and analytics engine, is a Java-based NoSQL database based on Apache Lucene^34^. With the help of Elasticsearch, organizations can retain, explore, and analyze massive amounts of data fast and in close to real-time, with results arriving in fractions of a second. It examines an index rather than the text itself, which enables it to deliver quick search results. Instead of using records and databases, it employs a framework based on documents and has robust REST APIs (Representational State Transfer Application Programming Interface^35^) for maintaining and accessing the data. Elasticsearch can be conceptualized as a server that can respond to JSON requests by providing JSON data. Elasticsearch data is visualized and the content is explored using the free and open-source Kibana interface. For instance, because Kibana is frequently used for log analysis, it is utilized to find out information about dissemination URLs, web hits sources, etc. The visualization of data can be commenced with one query and figured out as per requirement through interaction. A significant constraint, however, is the fact that each visualization should only be used with a specific index or indexing sequence. If an index has data that is completely different from another index, then distinct visual representations for each index must be created. Figure 3 depicts an overview of the experimental workflow.

### Overview of deployed honeypots

The system implementation provides the following images for various docker honeypots. Cowrie^36–38^, a medium interaction to high interaction SSH and Telnet honeypot that is the successor of the Kippo Honeypot. It is designed in Python and includes a flexible fraudulent file system, and also a falsified shell. Additionally, it is able to copy files via SFTP (SSH File Transfer Protocol) for subsequent analysis. In high interaction (Proxy mode), it functions as an SSH as well as telnet proxy to track the actions of the assailant with another system. Under medium interaction (Shell mode), it imitates a UNIX framework in Py. In the most recent version, Cowrie adds TCP/IP tunneling to capture proxy requests and the associated data. Every interaction is recorded in JSON format, and every session that is made is archived.

For the purpose of tracking and observing Multicast (UDP) Distributed Denial of Service (DDoS) assaults, the DDoSPot honeypot is deployed. It is an UDP-based platform that acts as a booby trap that aids to monitor and track DDoS attacks. The mentioned facilities or honeypot servers are supported by the system in the form of straightforward plugins referred to as pots: DNS (Domain Name System) server, NTP (Network Time Protocol) server, SSDP (Simple Service Discovery Protocol) server, CHAREN (Character Generator) server (port 19), and Random or mock UDP server. Ports 19, 123, 1900, and 53 are advertised as being used for communication.

Dionaea, a low interaction honeypot^38–40^ that simulates and offers a wide range of protocols, including FTP (File Transfer Protocol), TFTP (Trivial FTP), HTTP (Hyper Text Transfer Protocol), HTTPS (HTTPSecure), MQTT (MQ Telemetry Transport), MSSQL (Microsoft SQL), MySQL, SIP(Session Initiation Protocol), SMB(Server Message Block), and UPnP(Universal Plug & Play). Dionaea uses the libemu library for x86 virtualization to collect suspicious activities. Heralding is a low interaction honeypot^38,41^ that mimics a number of access interfaces in order to record inputted credentials, and source or destination ports. It offers a set of protocols, including Telnet, SSH (Secure Shell), FTP, HTTP, POP (Post Office Protocol), SMTP (Simple Mail Transfer Protocol), etc.

ADBHoney honeypot is a low interaction honeypot for the TCP/IP ADB(Android Debug Bridge)^42^. The Android Debug Bridge (ADB) protocol monitors actual and emulation-based cellphones, televisions, and DVRs linked to a specific host. It includes a number of commands (such as *adb shell*, *adb push*, etc.) that help developers push information to the machine while troubleshooting.

To analyze web applications and provide the replies that are then served by SNARE, TANNER honeypot is utilized which renders remote analysis of data and categorization service. When offering replies for SNARE (Web app honeypot detector that draws all kinds of internet coercion), TANNER employs a variety of application vulnerability type emulation strategies. Additionally, TANNER gives dorks to SNARE so that it can power its luring skills. Redis Honeypot, is a high interaction honeypot that traps redis vulnerabilities. ReDiS (Remote Dictionary Server) is an in-memory key-value data store that persists on disc. All redis data resides in memory thus enabling high throughput and low latency^43^.

Log4J honeypot is an internal network low-interaction honeypot that aids in sighting internal threats or intruders who search your network for log4j. It merely keeps an eye out for strange text patterns in queries (in input fields or Http headers) that notify via messages if anything odd shows up. Mailoney, a low-interactive SMTP honeypot that can simulate many vulnerabilities such as open_realy (logs every effort to deliver an email), postfix_creds( logs credentials of login trials), schizo_open_relay( logs every activity).

Honeytrap^44^ honeypot serves as a trap for all communication in the setup that has not been caught by another honeypot. It keeps track of arriving frames and launches TCP and UDP observers so that information can be captured. Ciscoassa, low interaction honeypot for Cisco ASA ( Cisco Adaptive Security Appliance element that can find the DoS (Denial of Service) and remote code execution flaw known as CVE 2018-0101 (A flaw in the Cisco ASA (Adaptive Security Appliance) Software’s SSL Layer (Secure Socket Layer) VPN capabilities allow unauthenticated, remote potential attackers to force a system restart or remotely perform operations) has also been employed.

CitrixHoneypot identifies and records activities of CVE-2019-19781 exploit (CVE-2019-19781: Citrix Application Delivery Controller (previously known as NetScaler ADC), as well as Citrix Gateway (also known as NetScaler Gateway) have been found to include vulnerabilities that, if abused, might grant an unauthorized attacker access to execute the malicious script). The honeypot Glutton allows for the collection, recording, and analysis of forwarded traffic by serving as a gateway between an adversary and another honeypot. In general, it pays attention to every port and then operates in accordance with rules in *rules.yaml* (found in */root/tpotce/docker/glutton/dist/rules.yaml*) file. The predominant library used by the proxy to handle packages and carry out its functions is *Freki* (which orchestrates packets in userspace via NFQueue, a focus of iptables & ip6tables, that outsource the action on packets to a user mode application).

Elasticsearch, Kibana, and Logstash, which are all elements of the ELK Stack^45,46^, were used to analyze and visualize the honeypot data. Built on top of Apache Lucene, Elastic Search is released under the Apache license. For its decentralized search engine, it employs the REST (REpresentational State Transfer) architectural style. Kibana is an analytics software program that uses elastic search, data visualization, and input from other elastic stack elements. Subsequently, Logstash is utilized as an information gathering backend to make it simple and quick to integrate logging file storage and retrieval. The honeypots disclose a combination of well-known and uncommon ports. These include ports such as 80 and 443 (HTTP/HTTPS), port 21 (FTP), port 22 (SSH), and port 445 (SMB and Microsoft Active Directory). Port 64297 was used to give access to the dashboard using authentication mechanisms to the overall system and port 64295 for SSH access.

As decision-making tools for forensics and network surveillance, the honeypot cluster employs FATT (Fingerprint All The Things: built using pyshark, allowing the extraction of networking meta - data and trace evidence from real-time communication or datagram capturing pcap files. JA3 : TLS client or server fingerprinting, HASSH: SSH client or server fingerprinting, RDFP: RDP fingerprinting for RDP protocol guideline,etc. are the profiling techniques it integrates that aid to run threat intelligence techniques inside a communication network), p0f ( Passive Operating system Fingerprinting: Uncover the entities around any TCP/IP interaction (even a regular SYN message), purely silent flow fingerprinting without intervening in any form), and suricata. The folders */OPT/container_name/LOG* and */OPT/container_name/DL* of the container have logs and files uploaded to the honeypot . These paths are mapped to the host machine volumes */DATA/container_name/LOG*. Additionally, certain facilities offer the tools cockpit, cyberchef, elasticsearch head, spiderfoot, and suricata, that further aid in the management, tracking, and assessment of honeypot systems as well as the dissemination of findings to the public.

### Implementation

The honeypots were set up on Microsoft Azure Cloud in different regions which aid to perform an analysis of the demographics of attack vectors in diverse locations (region 1: Central India, region 2: East Japan). For the entire experiment, each instance operated Debian 11(bullseye-Gen1 image, vm instance-type E2ds_v4) and had two virtual CPUs, 16 gigabytes of memory, 128 gigabyte hard disc, and a static Ip address. The containers set up in the system operate in host-network mode, which shares the host system’s network virtualization stack hence no separate IP address is assigned. In total, the system was operational 24 hours a day for 10 days, from 27th June 2022 to 6th July 2022. In order to minimize the discrepancies brought on by week-to-week variations in the attack surface, each instance began and concluded in a synchronized manner. To expose the system to exploitation, all ports are left open for incoming traffic. This experimentation offers even more useful information that will extend cyber security to propel forward. We installed the honeypot by leveraging T-Pot installer on the cloud. Post installation prompt to create a password for the user tsec and / or create a *<web-username>* and *password* that will be used for subsequent execution. Furthermore, if *tpot.conf* file in the *tpotce/iso/installer* directory is customized, then the installer can run automatically.

## Analysis of the captured data

**Figure 4:**
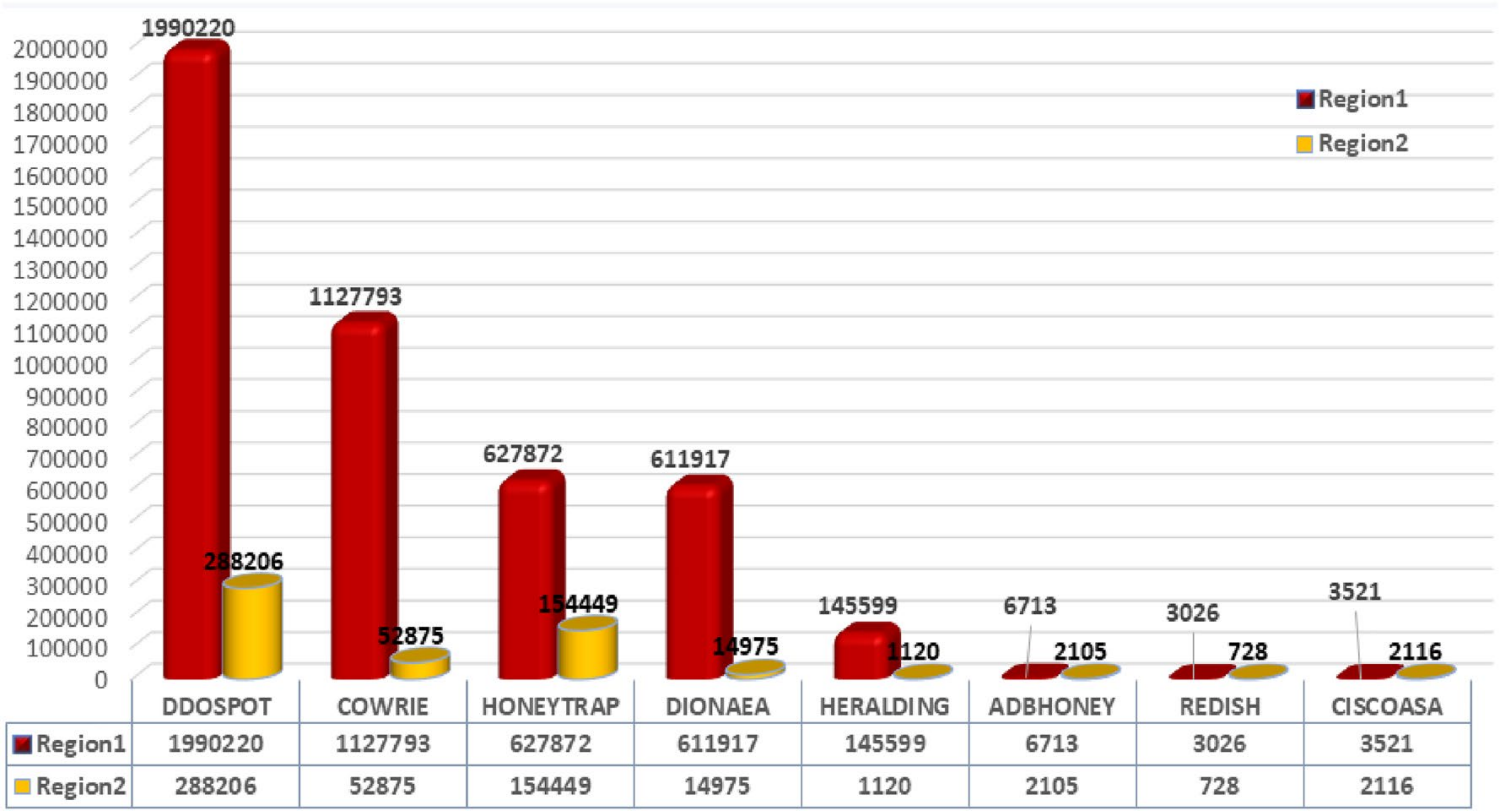
Attack statistics for Honeypot instances in experimental cloud geographies.

After setting up the cloud honeypot instances at two regions the front-end interface is examined at https://<*external IP address*>:64297, and used the credentials created during setup to log in. Figure 4 represents the overall attacks received in both regions. Attacks commenced arriving after the IP was made public on the internet. Knowing that you will experience assaults practically immediately after exposing an IP address makes it obvious that there would be bots or scans constantly searching cloud’s IP range. The same emphasizes how crucial it is to protect our cyber assets before enabling accessibility for operations. The attack statistics obtained by our experimentation are sketched in Fig. 4.

### Analysis of DDoS strikes

We executed a deep dive into DDOSPOT honeypot as it had been found to report the highest frequency of hits at both instances. A variety of automated tools and services offered by third-party suppliers for DDoS as a service make it simple to start DDoS attacks^47,48^. It is easy for even a beginner in attacking to initiate sophisticated DDoS assaults by using the key aspects of cloud technology.

**Figure 5:**
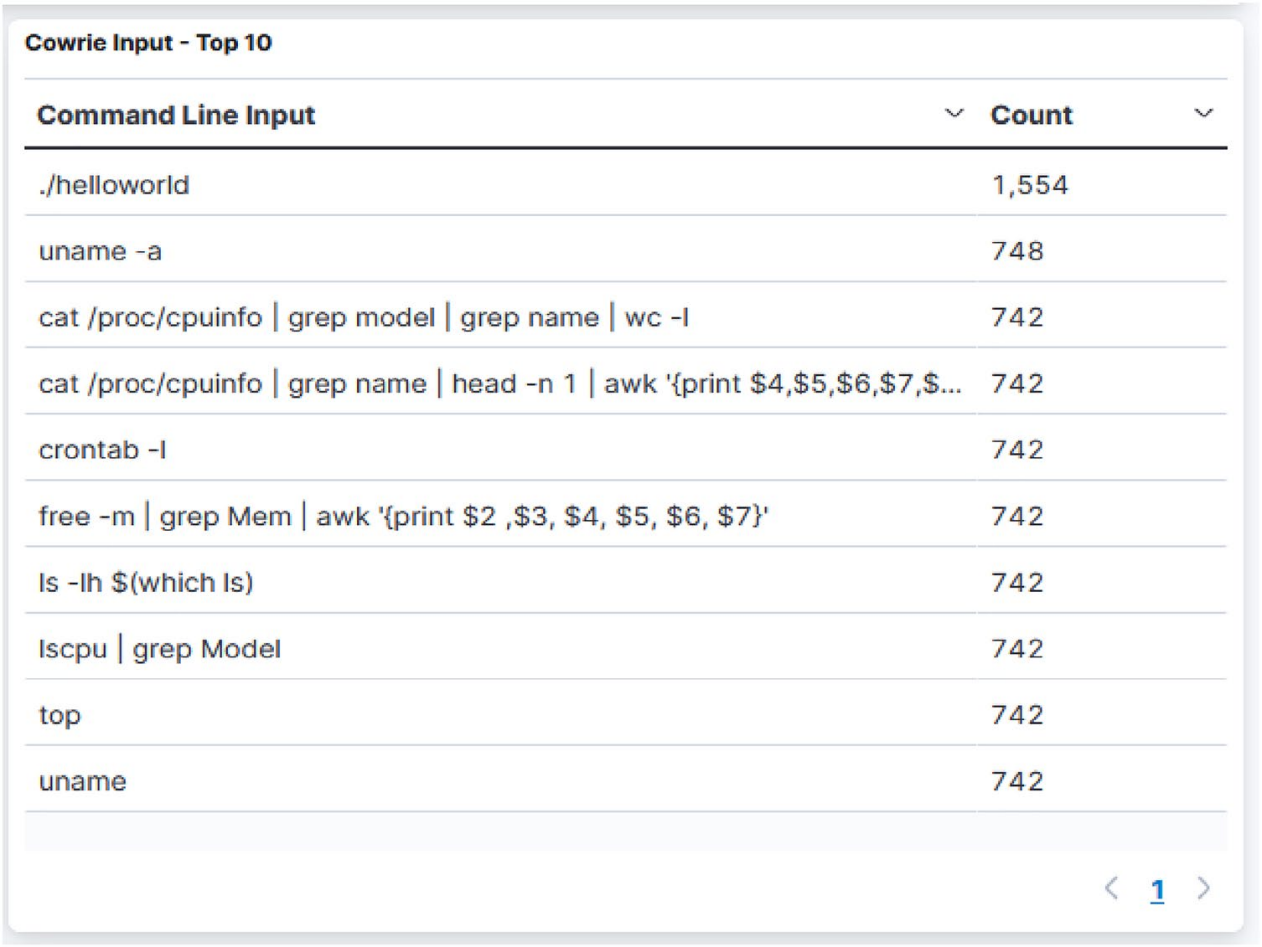
Sample commands executed by the adversaries upon accessing the system.

It’s not likely that every attack originates from an unidentified source using an unidentified IP . If an user launches any one of the cited commands, warnings may be set off by the majority of SIEMs (Security Information and Event Management). By doing this, you can establish policies to freeze the account until you receive assurance that there was no breach and perform any of those instructions. Building a defense necessitates comprehension of what adversaries undertake after gaining direct exposure. Figure 5 lists the frequently used commands by the malevolent user and Table 1 details it.

**Table 1:** Frequently utilized commands by the adversaries and their observations.

Commands	Observation
helloworld	Proof-of-concept for the possibility of executing arbitrary code through exploitation in which designers would not aim for the code to be executed
uname -a uname	Display system information like the name of the operating system, the name of the system node, the operating system release and its version, the name of the hardware, and the processor type
cat /proc/cpuinfo $$\mid$$ grep model $$\mid$$ grep name $$\mid$$ wc -l cat/proc/cpuinfo $$\mid$$ grep name $$\mid$$ head -n 1 $$\mid$$ awk print $4,$5,$6,$7,$8,$9	Display CPU information like its model, its name prints number of lines in the file ,prints the first CPU information details
ls -lh $(which is)	List the details of the files in the system, its permission type, created date and time in human-readable format

**Figure 6:**
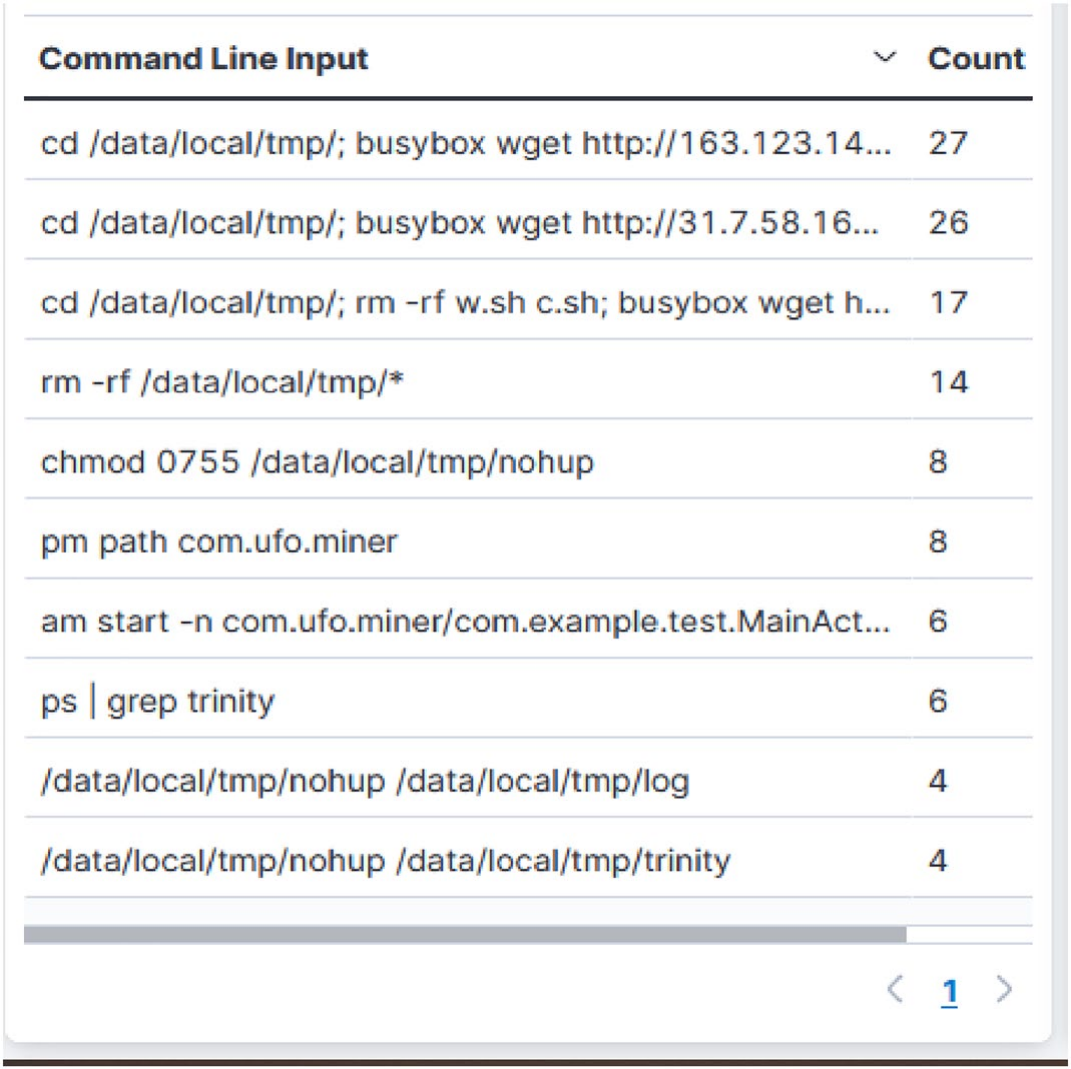
Commands executed by the adversaries upon access for launching busybox and trinity botnet attacks.

Analyzing the data acquired and using it as a point of reference for the auditing process, helps enterprises to better understand their existing security posture. Ddospot honeypot tagged known attacker, malware, tor exit node, bitcoin nodes, form spammer, mass scanner, anonymizer, bot, and crawler and of the total attacks received 71% of the attackers were known attackers that have already been outlined for engaging in despicable activities. We could pindown busybox wget vulnerability exploitation and trinity P2P malware attack (Fig. 6). Many-times, downloading an intermediary script that downloads the malware is preferred over downloading the infection directly. Hence attackers exploit the “busybox wget” vulnerability to download *w.sh* shell script from the IPs *31.7.58.162 and 163.123.142.144*; both tagged in blacklist as per^49^.

From the analysis of Fig. 6, when the device has been impacted with trinity malware attack, it is set up to search for all the other exploitable ones within the network having port 5555 open^50^. A shell is established and the command *“pm path com.ufo.miner”* is used to check if the malware is already mounted. If it has not, an apk file with the malicious programs will be sent to the entity, which will then be used to configure it and delete the installer. Following the miner’s installation, the apk’s *“am start -n com.ufo.miner/com.example.test.MainActivity”* command initiates the ufo bot. If the trinity bot is not already running, the command *“ps* $\Vert$ grep trinity” is performed, and if not, the *“trinity”*, *“nohup”*, and *“script”* files are installed and the *“tmp”* directory is cleared. Using *chmod*, the permissions on the nohup and trinity files are edited in such a way that all have read as well as execute permissions while the owner has read as well as write permissions. The Trinity botnet assault has the consequence of including affected machines in the botnet, which then continues to harvest cryptocurrencies for the assailant.

**Figure 7:**
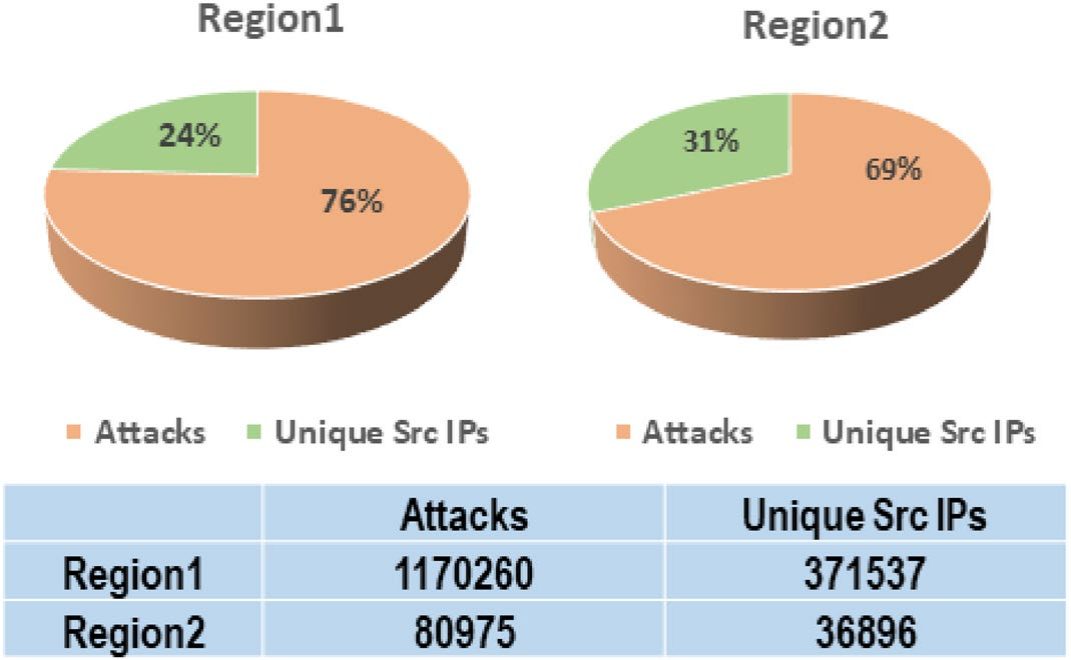
Unique source IP count by the Ddospot honeypot.

By tracking fluctuations in the number of distinct source ip addresses over the course of an event window and the number of source IPs with data transmission outstrips a specified threshold, the information on the number of distinct source IPs can be used as a DDoS attack detection component^51^. The count of distinct Src IP addresses and the number of non - complying source IP addresses can be assessed during each period of observation (Fig. 7) to look for security breaches. Both will deviate less from their mean values in normal circumstances, but during an attack, this deviation becomes more prominent.

**Figure 8:**
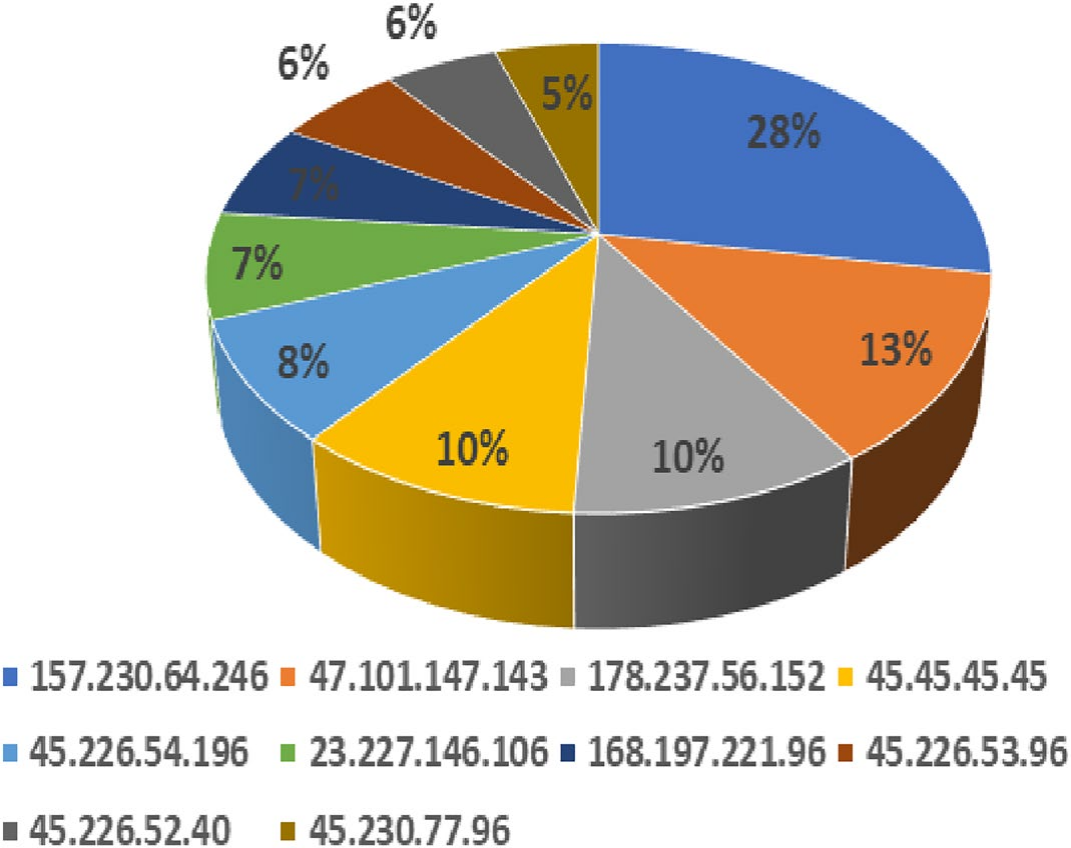
Attackers Source IP count percentage by Ddos honeypot.

Owing to the fact that the top four attackers on the list in region1 (Fig. 8) *157.230.64.246, 47.101.147.143, 178.237.56.152, 45.45.45.45* had more hits than others in the list, we prefer to concentrate our research on them. The IP reputation lookup from WhatIsMyIPAddress and DNSChecker designates DigitalOcean-ASN ISP * 157.230.64.246* as malicious as it reported spam score of 508.4^49^. The IP *47.101.147.143* from the network owner Hangzhou Alibaba Advertising Co.Ltd reported a critical spam level and have poor email reputation. The IP addresses 178.237.56.152 from Amsterdam, Netherlands (owner Hostcircle b.v.) and 45.45.45.45 from ISP Videotron Lee, Canada, have been flagged for IP and domain reputation issues by DNSBL(Domain Name System Blacklist: https://matrix.spfbl.net/ipaddress) and VirusTotal (https://virustotal.com). E-mails posted from these IPs have been either banned or sent to the recipient’s spam box, or treated accordingly. The remaining IP addresses are also listed on blocklists and are categorized as assailants.

### Suspicious URL commands

**Figure 9:**
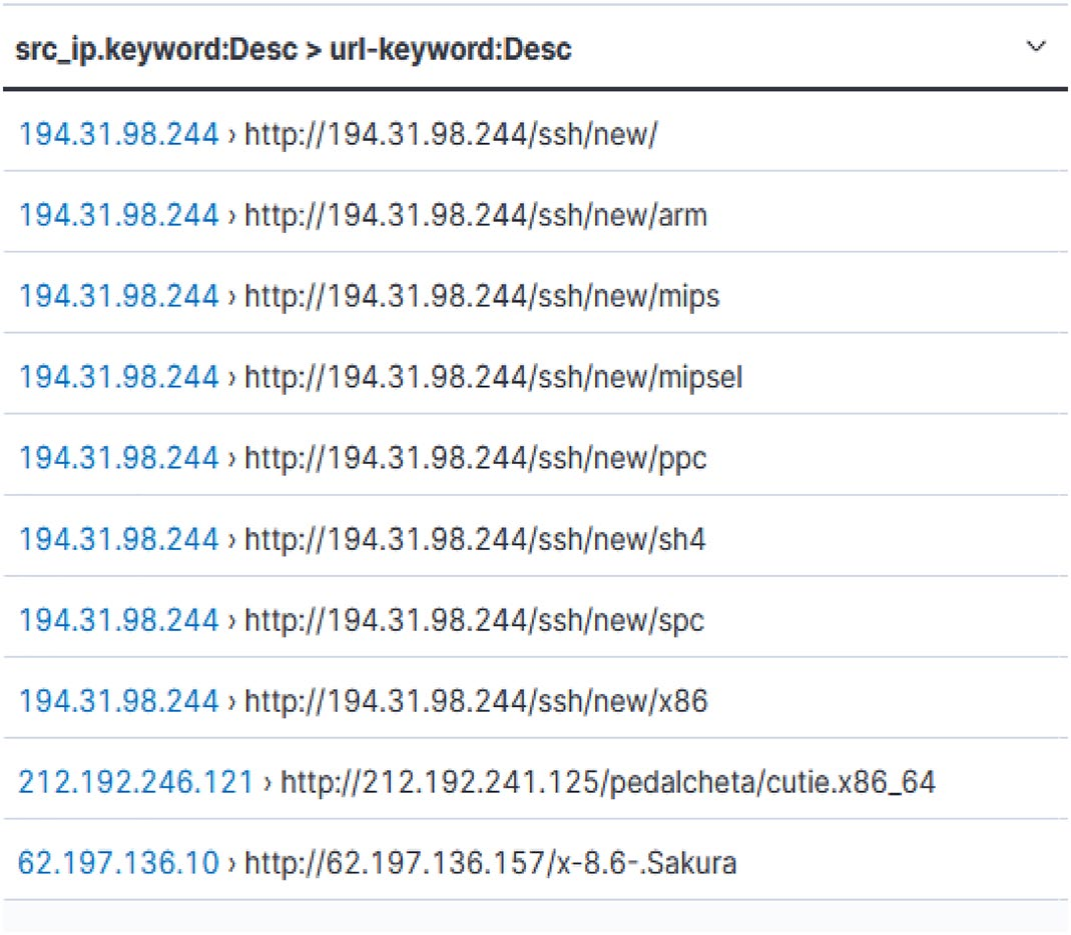
Suspicious URL commands.

Some of the intruders attempted to download malicious content from other sources after they gained access to our honeypot (Fig. 9). The details of the malicious URLs (ref: https://www.virustotal.com) has been listed in Table 2. SHA-256 column in the table denotes the body of the HTTP reply that the server provided back after being asked for the URL being looked up.

**Table 2:** Sample suspicious URLs with its details.

URL	SHA-256	No. of Vendors flagged	Response length
http://194.31.98.244/ssh/new	53d85d8c4ee63eca18604bc5db5f1ad732c789c18c03e1ef5462a1364aba1da1	17	169 B
http://194.31.98.244/ssh/new	aea0472eac4f3f62d5fb9273887351c1d992cb072222c819511f3f76f91be12f	16	85.43 KB
http://194.31.98.244/ssh/new/mips	1054d2bb71bde08a05581351d013460d2e3c5409e32a20245df5246e5dd43b8d	17	105.57 KB
http://194.31.98.244/ssh/new/mipsel	7d32537aeb584467a72b9981baa7a9ef4f9f0ec22da9c138423e9440d063db54	17	107.21 KB
http://194.31.98.244/ssh/new/ppc	b1e5fc0c284e4b731279af7c700e87572a938d50cd905cb9c2d45ddbc7ba124d	15	571 B
http://194.31.98.244/ssh/new/sh4	dd2943d2f8c69925d2c6248e82f232d5c75efca81b0b16d580773e2d890133b6	15	169 B
http://194.31.98.244/ssh/new/spc	1975851c916587e057fa5862884cbac3fa1e80881ddd062392486f5390c86865	15	97.13 KB
http://194.31.98.244/ssh/new/x86	ba04ee6e08b7688b6f2f8da9cb36f7e0c8facbf55b91c6170f7fa5d2f729d34a	16	83.58 KB
http://212.192.241.125/pedalcheta/cutie.x86_64	ae8dfd0c2f7e45d09a01d7982773628ae575ea535345ad18f51263ac162f3b50	14	66.84 KB

### Auxillary analysis

**Figure 10:**
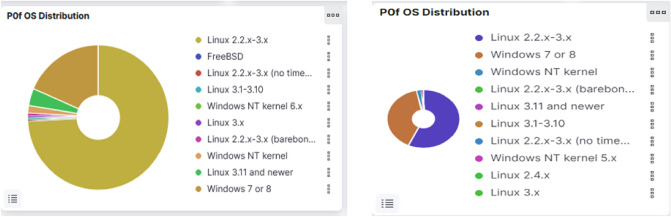
List of operating systems utilized by the intruders for launching invasion.

P0f that performs passive operating system detection using SYN packets aids in identifying the operating systems prevalent on the devices used by attackers. Distinct operating systems are employed to carry out strikes, mostly from Linux 2.2.x–3.x OSs and Windows 7 or 8 OSs as shown in Fig. 10. Linux is a popular operating system option for hackers for its adaptability, open source nature, portability, command-line interface, and interoperability including well hacking tools.

**Figure 11:**
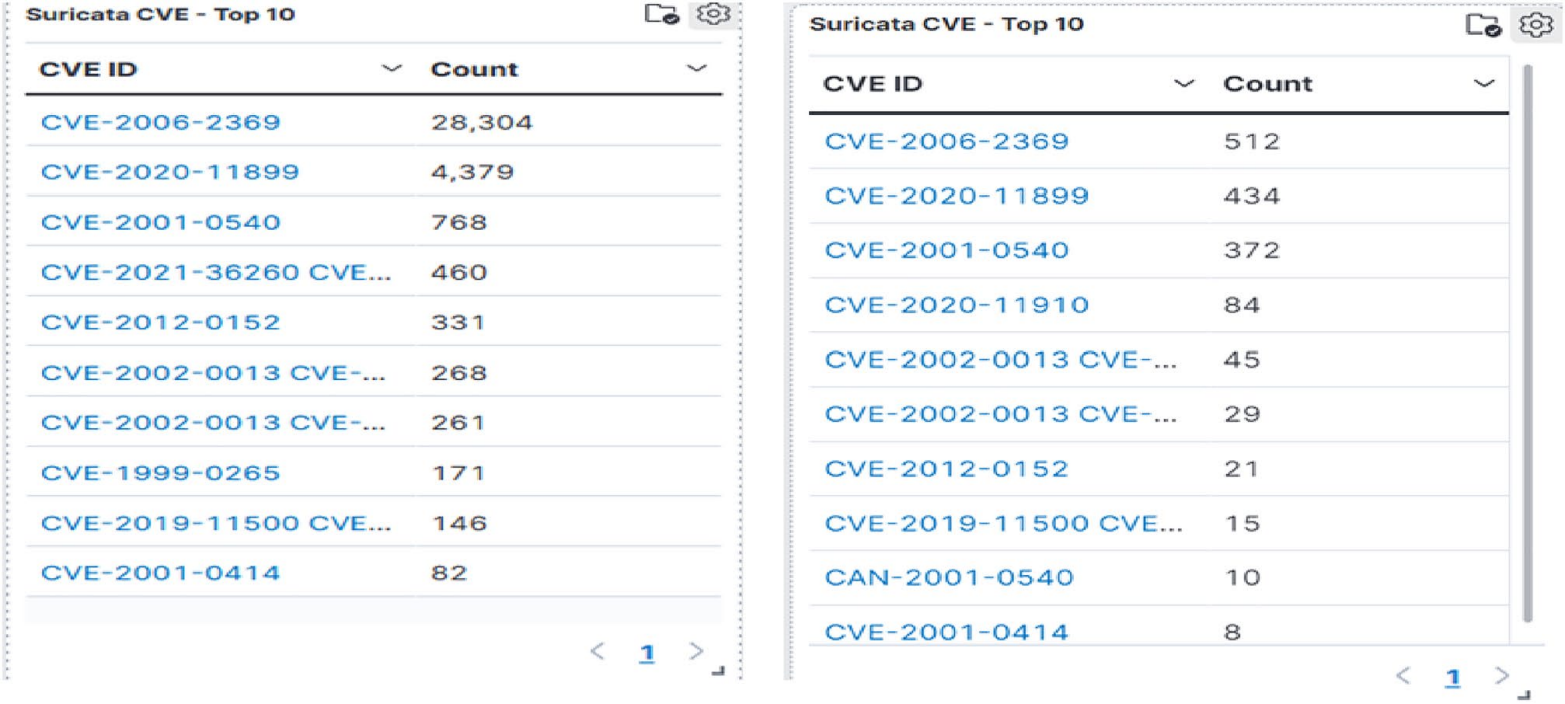
Details of top CVEs identified by the honeypot system.

Suricata CVE data is another resourceful element in the interface. CVE identification is offered by the advanced threat engine Suricata^52,53^. Recent CVEs being exploited are CVE-2021-36260(web server flaw: across several Hikvision appliances that involves command injection. Attackers can use the flaw to execute a command injection assault by delivering some messages that include malicious commands since there is inadequate input authentication), CVE-2020-11899 (Treck TCP/IP stack exploit: incorrect input validation when accepting a packet sent by an illegal access attacker in an IPv6 component. This flaw could lead to an out-of-bounds read as well as a Denial of Service). It is obvious that attackers are still using the prior flaws (Fig. 11) and hence organizations should address those deficiencies at the earliest.

**Figure 12:**
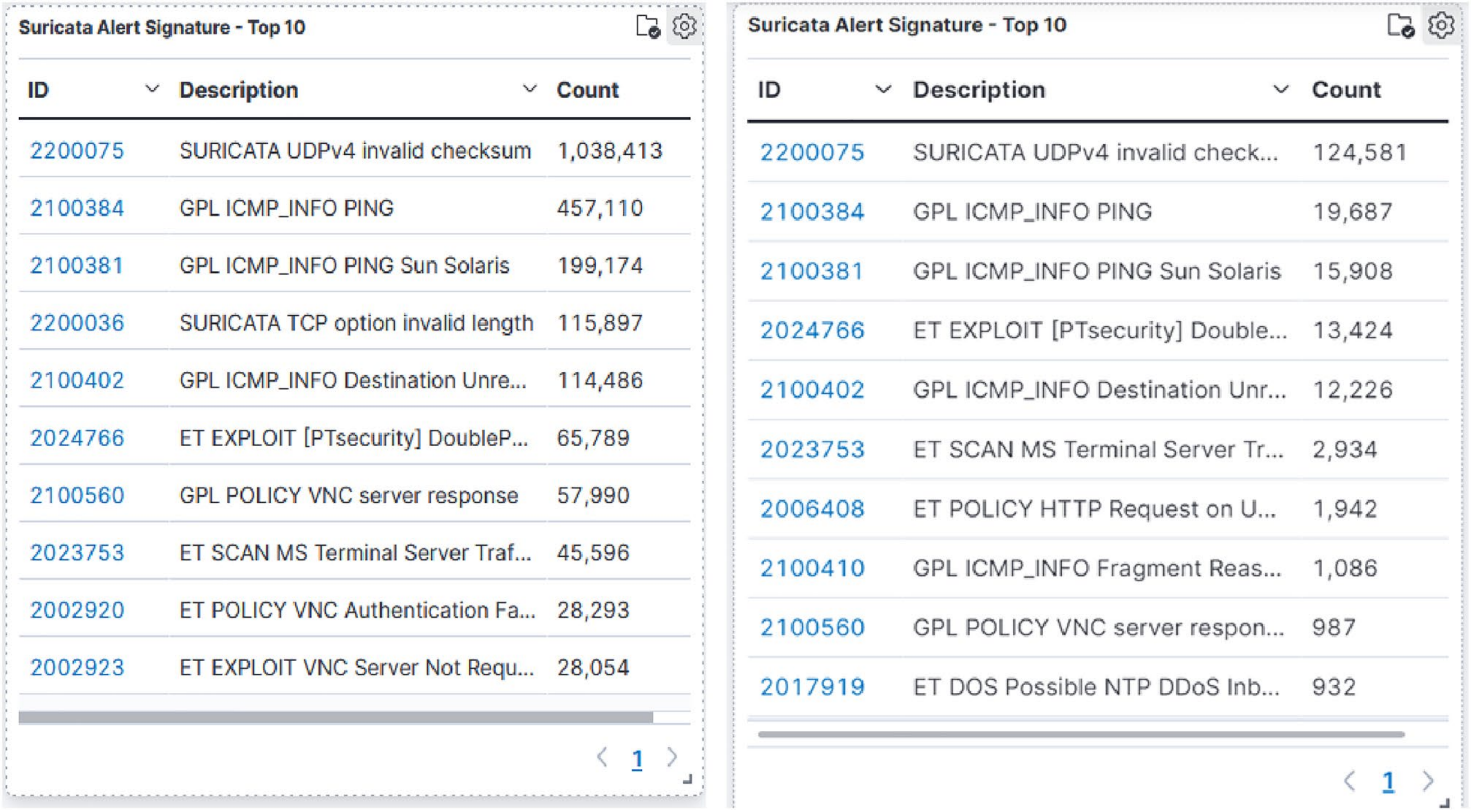
Details of alert signatures identified by the honeypot system.

*ICMP_INFO PING* & *UDPv4 invalid checksum*, are the highly reported alert signature by suricata as shown in Fig. 12. *ICMP_INFO PING* reveals that ping is still the widely used reconnaissance tool used by hackers to determine whether the specific host is reachable via an IP network or not. In UDP, no assurance that the packets will be received at the other end. However, if they are indeed delivered, are examined and dropped if they mismatch the checksum (*UDPv4 invalid checksum*). A User Datagram Protocol (UDP) overflow is a type of massive DoS attack in which the attacker hits and flushes arbitrary ports on the target with IPv4 packets that contain UDP packets^54^. This indeed results ultimately contributes to significant hits in the DDoS honeypot.

Hits reveal that the overwhelming attacks target SMB on port 445. This is the service that many exploit, including the more current SMBBleed/SMBGhost and Eternal Blue/WannaCry (*CVE-2017-0144*), (*CVE-2020-1206*). All throughout the world, strikes were reported, with the typical offenders being named^55^. This information can be used to carefully restrict access to those regions’ ASNs and IP addresses for vital services. The interesting fact that there were no hits on the Log4j honeypot could be attributed to the organizations being highly informed and upgrading to a patched Log4j version.

## Conclusion and future enhancements

Despite not being a cutting-edge technology, honeypots appear to significantly increase the volume of information that an enterprise can harvest. Whilst honeypot information security would aid in the mapping of the threat landscape, they are equipped in seeing activities aimed only at honeypot. We cannot simply say that a threat doesn’t exist just because it hasn’t been addressed at the honeypot.

We were successful in operationalizing a quick polymeric system that offers researchers a great deal of information while keeping in mind our primary objective for the work, that of a real-time warning asset. The honeypots performed effectively in obtaining high associations and a lot of samples of malware that were significant during our experiment. We could also catch events for DDoS attack which was predominant. System setup offered a great all-in-one solution to commence tracking actual attacks on numerous services in real-time. The front-end monitors offered a fantastic approach for analyzing and organizing patterns among attacks and creating visually striking and potent perspectives from the front line of the web. Several honeypot frameworks have been proposed merely as proofs of concepts, and their designers no more provide assistance for them. Long-term ventures are infrequent. Works in the future can utilize the knowledge gathered from the system to investigate zero-day vulnerabilities and develop IP delisting and intrusion detection and prevention system policies. In our forthcoming revision, we envisage addressing the failure of the system to give an early warning when DDoS traffic is spotted by integrating intelligence.

## Data Availability

The datasets generated and analyzed during the current study are available at the link: RAW DATA.

## References

[1] Dwivedi, R. K. & Kumar, R. Sensor cloud: Integrating wireless sensor networks with cloud computing. in *2018 5th IEEE Uttar Pradesh Section International Conference on Electrical, Electronics and Computer Engineering (UPCON)*. 1–6. 10.1109/UPCON.2018.8597008 (IEEE, 2018).

[2] Baykara M, Das R (2018). A novel honeypot based security approach for real-time intrusion detection and prevention systems. J. Inf. Secur. Appl..

[3] *Ibm Report: How Much Does a Data Breach Cost in 2022*? Accessed 08 Dec 2022 (2022).

[4] Northport. N.Y. *Cybercrime Magazine* (2021).

[5] Batchu RK, Seetha H (2021). A generalized machine learning model for DDoS attacks detection using hybrid feature selection and hyperparameter tuning. Comput. Netw..

[6] Halvorsen J, Waite J, Hahn A (2019). Evaluating the observability of network security monitoring strategies with tomato. IEEE Access.

[7] Kumar, R.S.S., Wicker, A. & Swann, M. Practical machine learning for cloud intrusion detection: Challenges and the way forward. in *Proceedings of the 10th ACM Workshop on Artificial Intelligence and Security*. 81–90 (2017).

[8] Agrawal N, Tapaswi S (2017). The performance analysis of honeypot based intrusion detection system for wireless network. Int. J. Wirel. Inf. Netw..

[9] Shafiq M, Tian Z, Sun Y, Du X, Guizani M (2020). Selection of effective machine learning algorithm and Bot–IoT attacks traffic identification for internet of things in smart city. Future Gener. Comput. Syst..

[10] Shafiq M, Tian Z, Bashir AK, Du X, Guizani M (2021). Corrauc: A malicious Bot–IoT traffic detection method in IoT network using machine-learning techniques. IEEE Internet Things J..

[11] Shafiq M, Tian Z, Bashir AK, Du X, Guizani M (2020). Iot malicious traffic identification using wrapper-based feature selection mechanisms. Comput. Secur..

[12] Baykara M, Das R (2018). A novel honeypot based security approach for real-time intrusion detection and prevention systems. J. Inf. Secur. Appl..

[13] Artail H, Safa H, Sraj M, Kuwatly I, Al-Masri Z (2006). A hybrid honeypot framework for improving intrusion detection systems in protecting organizational networks. Comput. Secur..

[14] Sharma S, Kaul A (2018). A survey on intrusion detection systems and honeypot based proactive security mechanisms in VANETS and VANET cloud. Vehic. Commun..

[15] Kambow N, Passi LK (2014). Honeypots: The need of network security. Int. J. Comput. Sci. Inf. Technol..

[16] *Github: Ghost-usb-Honeypot*. Accessed 30 Sep 2021 (2021).

[17] Franco J, Aris A, Canberk B, Uluagac AS (2021). A survey of honeypots and honeynets for internet of things, industrial internet of things, and cyber-physical systems. IEEE Commun. Surv. Tutorials.

[18] Krishnaveni S, Prabakaran S, Sivamohan S (2018). A survey on honeypot and honeynet systems for intrusion detection in cloud environment. J. Comput. Theor. Nanosci..

[19] Fan W, Du Z, Fernández D, Villagrá VA (2018). Enabling an anatomic view to investigate honeypot systems: A survey. IEEE Syst. J..

[20] Wan X, Guan X, Wang T, Bai G, Choi B-Y (2018). Application deployment using microservice and docker containers: Framework and optimization. J. Netw. Comput. Appl..

[21] Pahl, C., Jamshidi, P. & Zimmermann, O. Microservices and containers. in *Software Engineering 2020* (Felderer, M., Hasselbring, W., Rabiser, R. & Jung, R. eds.). 115–116. 10.18420/SE2020_34 (Gesellschaft für Informatik e.V., 2020).

[22] Liu, G. *et al.* Microservices: Architecture, container, and challenges. in *2020 IEEE 20th International Conference on Software Quality, Reliability and Security Companion (QRS-C)*. 629–635. 10.1109/QRS-C51114.2020.00107 (2020).

[23] Rashid, S. *et al.* Faking smart industry: Exploring cyber-threat landscape deploying cloud-based honeypot. *Wirel. Netw.* 1–15 (2022).

[24] *The Honeynet Project: Spam Honeypot with Intelligent Virtual Analyzer*. Accessed 15 June 2022 (2022).

[25] *Liston, tom:labera*. Accessed 13 June 2022 (2022).

[26] Valicek, M., Schramm, G., Pirker, M. & Schrittwieser, S. Creation and integration of remote high interaction honeypots. in *2017 International Conference on Software Security and Assurance (ICSSA)*. 50–55. 10.1109/ICSSA.2017.21 (2017).

[27] Sun Y (2020). Honeypot identification in softwarized industrial cyber-physical systems. IEEE Trans. Ind. Inform..

[28] Tsikerdekis, M., Zeadally, S., Schlesener, A. & Sklavos, N. Approaches for preventing honeypot detection and compromise. in *2018 Global Information Infrastructure and Networking Symposium (GIIS)*. 1–6. 10.1109/GIIS.2018.8635603 (2018).

[29] Sun Y, Tian Z, Li M, Zhu C, Guizani N (2020). Automated attack and defense framework toward 5g security. IEEE Netw..

[30] Luo C (2020). A novel web attack detection system for internet of things via ensemble classification. IEEE Trans. Ind. Inform..

[31] Eibes, M. *Telekom Security*. Accessed 23 Apr 2022 (2015).

[32] *Elastic: Filebeat Overview*. Accessed 18 Dec 2022 (2022).

[33] Chen, L., Liu, J., Xian, M. & Wang, H. Docker container log collection and analysis system based on elk. in *2020 International Conference on Computer Information and Big Data Applications (CIBDA)*. 317–320. 10.1109/CIBDA50819.2020.00078 (2020).

[34] *Elasticsearch, B. Elasticsearch*. *https://www. elastic. co/pt/*. Accessed 12 Sep 2019 (2018).

[35] Arcuri A (2019). Restful API automated test case generation with EvoMaster. ACM Trans. Softw. Eng. Methodol. (TOSEM).

[36] Cabral, W., Valli, C., Sikos, L. & Wakeling, S. Review and analysis of cowrie artefacts and their potential to be used deceptively. in *2019 International Conference on Computational Science and Computational Intelligence (CSCI)*. 166–171. 10.1109/CSCI49370.2019.00035 (2019).

[37] *Github:cowrie/cowrie*. Accessed 22 May 2022 (2022).

[38] Kelly C, Pitropakis N, Mylonas A, McKeown S, Buchanan WJ (2021). A comparative analysis of honeypots on different cloud platforms. Sensors.

[39] Ali, P. D. & Kumar, T. G. Malware capturing and detection in dionaea honeypot. in *2017 Innovations in Power and Advanced Computing Technologies (i-PACT)*. 1–5. 10.1109/IPACT.2017.8245158 (2017).

[40] *Dinotools/dionaea*. Accessed 21 Jan 2022 (2022).

[41] *Github:johnnykv/herlading*. Accessed 24 Mar 2022 (2022).

[42] *Github:huuck/adbhoney*. Accessed 27 Jan 2022 (2022).

[43] *Nist:-nvd*. Accessed 27 May 2022 (2022).

[44] *The Honeynet Project*. Accessed 27 May 2022 (2022).

[45] Shah N, Willick D, Mago V (2018). A framework for social media data analytics using Elasticsearch and Kibana. Wirel. Netw..

[46] Azarmi B (2017). Learning Kibana 5.0.

[47] Agrawal N, Tapaswi S (2019). Defense mechanisms against DDoS attacks in a cloud computing environment: State-of-the-art and research challenges. IEEE Commun. Surv. Tutorials.

[48] Batchu RK, Seetha H (2022). A hybrid detection system for DDoS attacks based on deep sparse autoencoder and light gradient boost machine. J. Inf. Knowl. Manag..

[49] *Dns Checker; whatismyipaddress.com*. Accessed 20 Jul 2022 (2022).

[50] Cirlig, G. *Trinity-p2p Malware Over adb*. Accessed 21 Jul 2022 (2020).

[51] Baishya RC, Hoque N, Bhattacharyya DK (2017). DDoS attack detection using unique source IP deviation. Int. J. Netw. Secur..

[52] Nam, K. & Kim, K. A study on SDN security enhancement using open source IDS/IPS Suricata. in *2018 International Conference on Information and Communication Technology Convergence (ICTC)*. 1124–1126. 10.1109/ICTC.2018.8539455 (2018).

[53] *Nvd:cve*. Accessed 25 May 2022 (2022).

[54] Qiao, S., Hu, C., Guan, X. & Zou, J. Taming the flow table overflow in openflow switch. in *Proceedings of the 2016 ACM SIGCOMM Conference*. 591–592 (2016).

[55] Batchu, R.K. & Seetha, H. On improving the performance of DDoS attack detection system. *Microprocess. Microsyst.* 104571 (2022).

